# Respiratory syncytial virus infection in infants and correlation with meteorological factors and air pollutants

**DOI:** 10.1186/1824-7288-39-1

**Published:** 2013-01-11

**Authors:** Silvia Vandini, Luigi Corvaglia, Rosina Alessandroni, Giulia Aquilano, Concetta Marsico, Marica Spinelli, Marcello Lanari, Giacomo Faldella

**Affiliations:** 1 Neonatology -S. Orsola-Malpighi Hospital-University of Bologna, Via Massarenti 11, Bologna, 40138, Italy

**Keywords:** Respitatory syncytial virus, Bronchiolitis, Temperature, Humidity, Air pollution

## Abstract

**Background:**

Respiratory Syncytial Virus (RSV) is the most important cause of severe respiratory infections in infants with seasonal epidemics. Environmental factors (temperature, humidity, air pollution) could influence RSV epidemics through their effects on virus activity and diffusion.

**Methods:**

We conducted a retrospective study on a paediatric population who referred to our Paediatric Emergency Unit in order to analyze the correlation between weekly incidence of RSV positive cases during winter season in Bologna and meteorological factors and air pollutants concentration.

**Results:**

We observed a significant correlation between the incidence of RSV infections and the mean minimum temperature registered during the same week and the previous weeks.

The weekly number of RSV positive cases was also correlated to the mean PM_10_ concentration of the week before.

**Conclusions:**

RSV epidemic trend in Bologna (Italy) is related to the mean minimum temperature, and the mean PM_10_ concentration.

## Background

Respiratory Syncytial Virus (RSV) is the most important cause of severe respiratory infections in infants. In temperate climates, RSV infection typically has a seasonal trend and peaks during winter. Some authors [1-3] identified a correlation between RSV epidemic and climatic factors (temperature, relative and absolute humidity and UV-B radiation). Meerhoff et al [1]. analyzed RSV epidemics in different winter seasons and observed a correlation between RSV activity and relative humidity, minimum temperature and cloud cover in the Netherlands, with the effect of relative humidity being more consistent. Yusuf et al [2]. analyzed RSV activity in nine cities with different meteorological characteristics and observed a peak during winter in temperate climates, while in colder regions virus activity was nearly continuous. Moreover, some authors [4-7] reported a correlation between air pollutants (Particulare Matter < 10 μm [PM_10_, Particulate Matter <5 μm [PM_2.5_, Carbon Monoxide [CO], Nitric Oxide [NO_2_) and respiratory tract infections and RSV infections [8,9].

To our knowledge no authors examined the correlation between RSV activity and climatic factors in Italy.

Bologna is a city in the northern temperate area cold and cloudy during winter; January is usually the coldest month of the year and the temperature is higher in autumn than in spring.

Relative humidity presents less variable mean values through different months, ranging between 65% registered in July and 84% in November and December (mean values of the last 30 years). Bologna has almost 400.000 inhabitants with only two hospitals with a Paediatric Emergency Unit. An air pollution monitoring network has been in active since many years in the city area. Approximately 20.000 children are admitted to the Pediatric Emergency Unit of S.Orsola-Malpighi Hospital each year with approximately 1600 infants younger than 2 years referring to the Paediatric Emergency Unit during the winter season for acute respiratory tract infections.

RSV epidemic in Italy presents a seasonal pattern with onset in November, peak in February or March and offset in April [10].

We examined the incidence of RSV infections in children admitted to the Paediatric Emergency Unit of S.Orsola-Malpighi Hospital and we hypothesized a correlation with climatic factors and air pollutants concentration recorded during different winter seasons in Bologna.

## Methods

All patients younger than 2 years who referred to Paediatric Emergency Unit of S.Orsola-Malpighi Hospital of Bologna (Italy) for suspected acute RSV infection during 3 consecutive winter seasons from September to April (2007–2008, 2008–2009, 2009–2010) were included.

RSV infection was suspected in presence of fever, cough, respiratory distress with tachypnea, cyanosis, feeding difficulties; RSV rapid test was performed on nasopharyngeal secretions to all infants admitted to the unit presenting these symptoms.

Every week we recorded the number of infants who were tested with BINAX NOW, a rapid immunochromatographic assay for the qualitative detection of RSV fusion protein antigen in nasopharyngeal secretions.

Meteorological data of the geographic area of Bologna (minimum temperature,°C; relative humidity, %) were recorded from Bologna Borgo Panigale meteorological station; data were summarized as mean values for each week.

Air pollution data (PM_10_ and PM_2,5_ mean weekly concentration) of the same area were recorded from Bologna San Felice monitoring station by Bologna Department of the Regional Environmental Protection Agency (ARPA).

Ethics approval was not required for this observational and anonymous study. Consent was obtained for all patients.

Statistical analysis was performed with Microsoft Excel 2010. Pearson’s correlation was used to correlate the weekly number of RSV positive cases with meteorological parameters (mean minimum temperature, mean relative humidity) and air pollutants (PM_10_ and PM_2,5_) mean concentrations. A value of r>0,3 was considered statistically significant.

The number of RSV infections was also correlated to the climatic parameters of the same week (no lag), or one (1-week lag), two (2-week lags) or three (3-week lag) weeks before, aiming to identify a delay in the effect of temperature or humidity on RSV epidemics.

Considering air pollutants, we correlate the weekly number of RSV infections with the mean weekly concentration (μg/m^3^) of PM_10_ and PM_2,5_ measured in the same week (no lag) and in the previous week (1-week lag) aiming to enlight the short-term effect of air pollutants exposure.

## Results

Meteorological parameters and RSV activity during the three seasons are summarized in Table 1.

**Table 1:** RSV activity and meteorological variables by winter season for the period 2007-2010

**Year**	**RSV cases (n)**	**Wk onset**	**Wk peak**	**Wk offset**	**Minimum Temperature (°C)**		**Relative humidity (%)**	
					**Onset**	**Peak**	**Onset**	**Peak**
2007-08	125	46	1	16	1,5	−0,1	65	85
2008-09	74	45	7	16	11	−1,4	85	55
2009-10	128	1	8	16	0.5	5,1	93	83

Mean relative humidity and mean minimum temperature were similar during the three seasons; temperature was usually lower around RSV peak, while relative humidity remained more stable than temperature during the whole season.

The peak occurred at a mean minimum temperature range of −1,4-5,1°C and at a mean relative humidity of 55-85%.

We considered as the onset of RSV activity the first week RSV was detected; patients younger than 2 years referring to Paediatric Emergency Unit with symptoms compatible with RSV infection were tested all the year round but tests resulted positive only during the epidemic periods summarized in Table 1.

**Figure 1:**
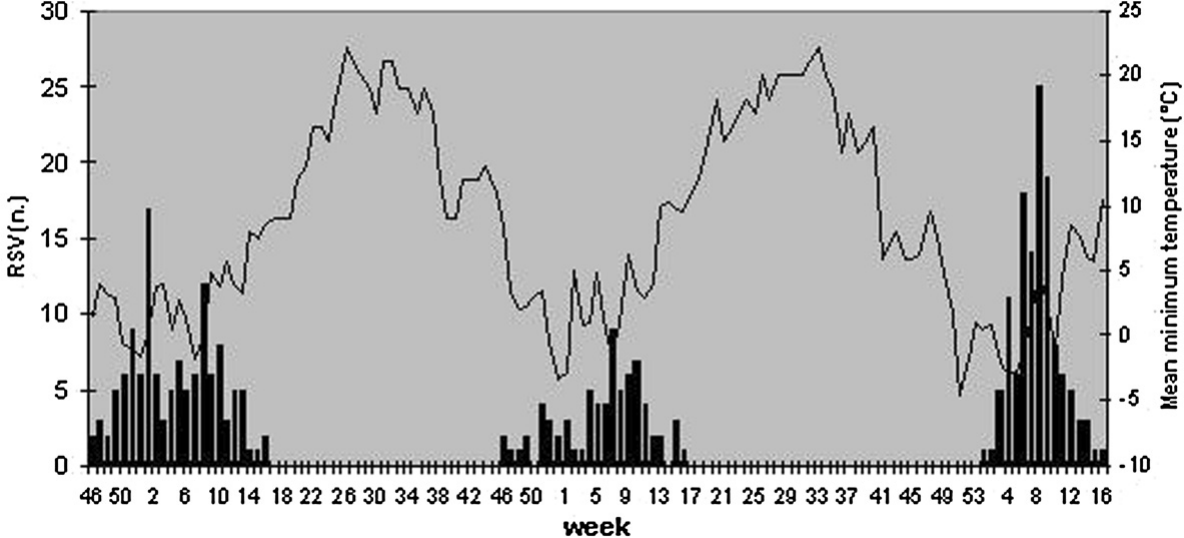
RSV positive detections and mean minimum temperature in the winter seasons 2007–2010 in Bologna.

**Figure 2:**
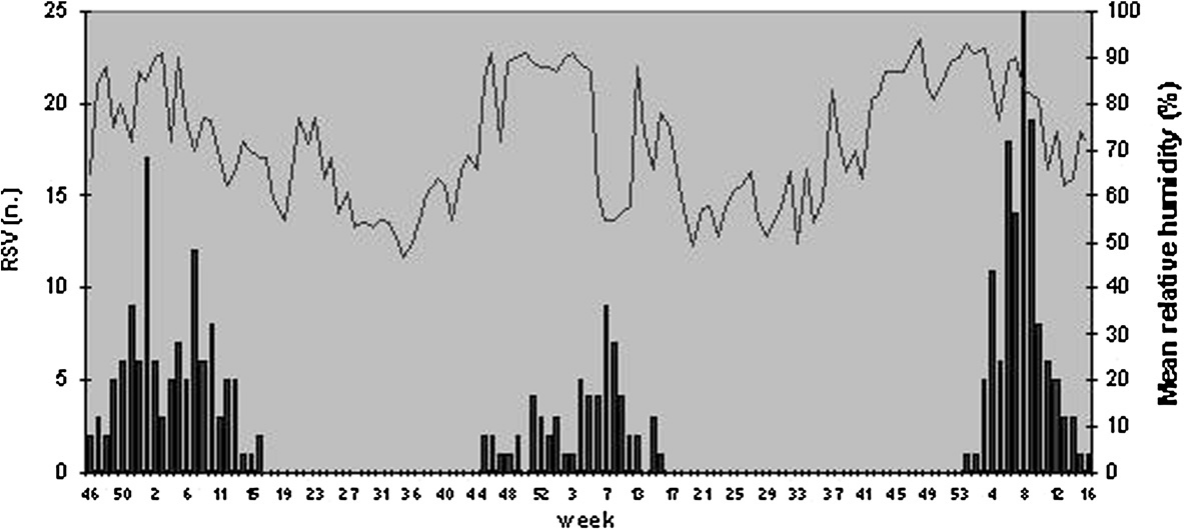
RSV positive detections and mean relative humidity in the winter seasons 2007–2010 in Bologna.

The onset of RSV activity in the three seasons occurred at week 46, 45 and 1 respectively, with peak activity at week 1, 7 and 8, as shown in Figures 1 and 2.

Pearson’s correlation showed a negative correlation between the number of RSV positive cases and the mean minimum temperature (r >−0,3) at different time lags as shown in Table 2; the correlation was not observed for the mean relative humidity.

**Table 2:** Pearson’s correlation between number of RSV detections and mean minimum temperature and mean relative humidity for different time lags

**Mean minimum temperature**	**r**	**p value**	**Mean relative humidity**	**r**	**p value**
No lag	−0,3	0,012	No lag	−0,05	0,68
1 week lag	−0,39	0,001	1 week lag	−0,007	0,47
2 week lag	−0,43	0,0002	2 week lag	0,13	0,14
3 week lag	−0,53	<0,0001	3 week lag	0,17	0,08

**Figure 3:**
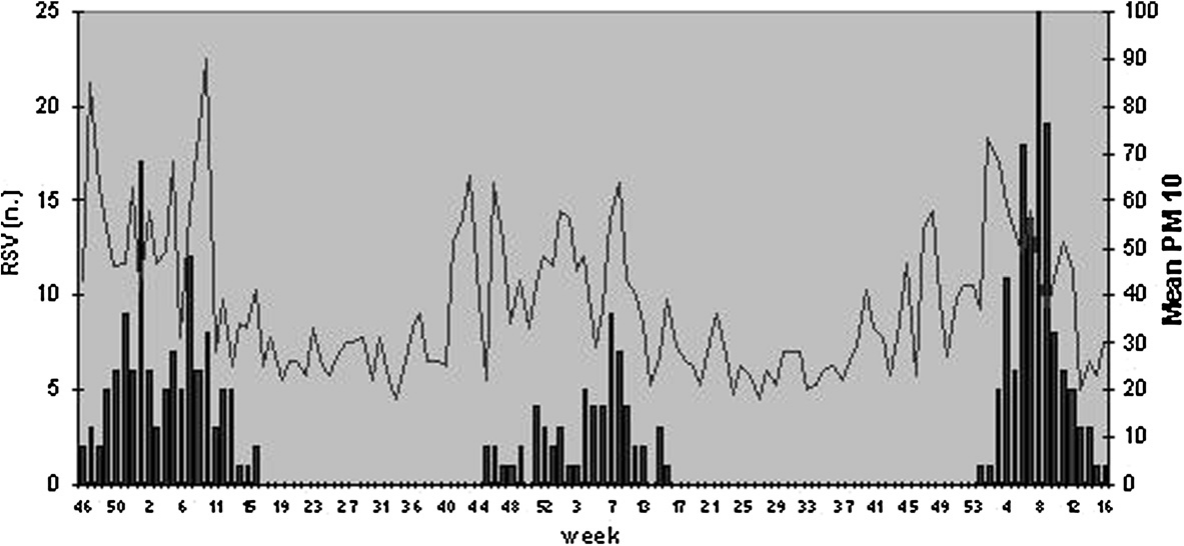
RSV positive detections and mean PM_10_ concentration (μg/m^3^) in the winter seasons 2007–2010 in Bologna.

**Figure 4:**
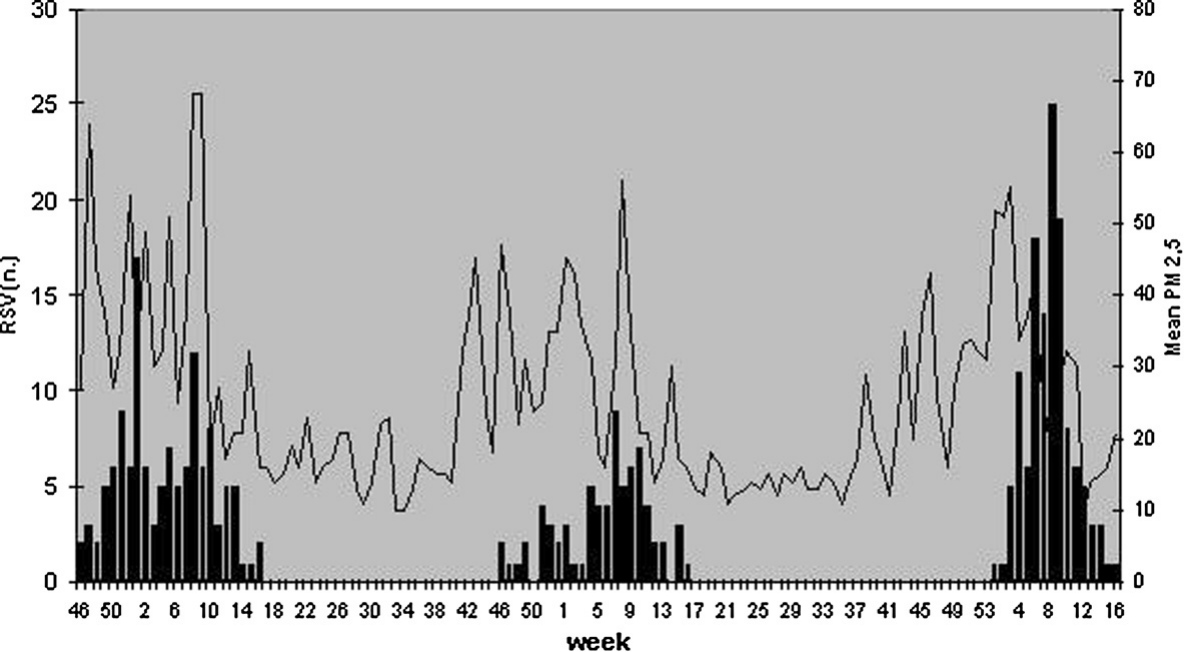
RSV positive detections and mean PM_2.5_ concentration (μg/m^3^) in the winter seasons 2007–2010 in Bologna.

The correlation between RSV detections and air pollutants concentration in the different time lags is summarized in Table 3. Pearson’s correlation between number of RSV positive cases and air pollutants showed a significant correlation (r=0,3) between RSV infections and PM_10_ mean concentration of the week before (Figure 3) a significant correlation between RSV infections and PM2.5 mean concentration was not found (Figure 4).

**Table 3:** Pearson’s correlation between number of RSV detections and mean concentration of air pollutants PM_10_ and PM_2,5_ for different time lags

**Mean PM**_**10**_**(μg/m**^**3**^**)**	**r**	**p value**	**Mean PM**_**2,5**_**(μg/m**^**3**^**)**	**r**	**p value**
No lag	0,2	0.051	No lag	0,14	0,25
1 week lag	0,34	0,004	1 week lag	0,26	0,03

## Discussion

RSV activity in Bologna presents the pattern described in temperate areas, with onset and peak during winter season. Meteorological conditions in the 4 weeks of peak activity for each season were similar to data presented by other authors [1-3].

These conditions are the mean minimum temperature 2-6°C and mean relative humidity 45-65% and are associated to the greatest activity of the virus [11-13].

In 2009–2010 the onset occurred later than the previous years; a delay in RSV epidemics during the season 2009–2010 has been observed in France by Casalegno et al [14]. These authors observed that RSV epidemic started more gradually with a delayed peak and hypothesized that this delay might be partially caused by pandemic H1N1 Influenza in 2009 through viral interference and increased hygiene measures.

A study performed in Croatia [15] presented a biennial cycle of RSV outbreaks with one large and one small season; the correlation between RSV epidemic and climatic factors was observed only for the larger season.

In the present study we cannot demonstrate the effects of other respiratory viruses on RSV delayed peak since we included only RSV detection which can be performed in a few minutes at Paediatric Emergency Unit to all infants with respiratory symptoms.

Moreover, a study period including several additional winter seasons would probably be warranted in order to determine the overall patterns of RSV epidemic onset in Bologna and Emilia-Romagna and to confirm whether a biennial cycle of RSV outbreak is detectable in our geographic area.

RSV activity is greater at cold temperature because the virus is more stable in secretions by which it is transmitted. Moreover, cold temperature might drive populations indoor where RSV spreads more readily.

The correlation between mean minimum temperature and RSV epidemic activity persists in the three different time lags. We hypothesize that a decrease in the mean minimum temperature may determine a greater RSV activity in the following weeks associated with more prolonged permanence indoor, sometimes in overcrowded rooms; these conditions may determine an increase in RSV diffusion, which is followed by viral incubation (2–7 days) and lead to an increase in number of RSV infection.

The weak relationship between RSV epidemics and relative humidity can be explained by the small variability and the irregular weekly pattern of this parameter during the whole winter season in Bologna. This result is different from data collected in other countries such as the Netherlands [1], where relative humidity has greater seasonal fluctuations and the effect of relative humidity is more consistent. We also analyzed the correlation between RSV detection and air pollutants concentration. Many authors [5-7,9] showed a positive correlation between fine particulate matter (PM_10_ and PM_2,5_) and morbidity for respiratory infections and other respiratory conditions such as asthma and chronic obstructive pulmonary disease. This correlation is explained by increased respiratory symptoms, reduced lung function and bronchial reactivity related to air pollution exposure.

Moreover, these effects are increased in paediatric population, especially young infants, because of their higher respiratory rate that increases air pollutants per kilogram of body weight exposure.

The correlation with RSV infection [8,9] was found both for acute exposure (one week) and for subchronic and chronic exposure (30–60 days). We observed a similar correlation in our study between the weekly number of RSV detections and the mean particulate matter concentration of the week before. We examined only acute air pollution exposure (considering PM_10_ and PM_2,5_ concentration in the same week or in the week before) and not subchronic and chronic exposure because we considered the whole population who referred to our Paediatric Emergency Unit without considering differences in their residential address and the distance from the air pollution monitoring station.

Furthermore, the reduction of air pollution in urban areas could lead to an improvement in infants morbidity as it could determine a reduction in respiratory symptoms and individual susceptibility to respiratory infection.

## Conclusions

Environmental data collected in Bologna during 3 winter seasons confirm a correlation between RSV seasonality and mean minimum temperature and mean PM_10_ concentration.

## Abbreviations

RSV: Respiratory Syncytial Virus; PM_10_: Particulate Matter measuring 10μm or less; PM_2,5_: Fine Particulate Matter with a diameter smaller than 2.5 μm or less.

## Competing interests

All authors declare they do not have any financial and non-financial competing interest.

## Authors’ contributions

ML participated in the design of the study and reviewed the manuscript; GF coordinated the study and collaborated to the draft of the manuscript; RA, GA, MS and CM collected data about RSV infections; LC performed statistical analysis; SV drafted the manuscript and prepared the database. All authors read and approved the final manuscript.
